# Cesium Lead Chloride as an Artificial Solid Electrolyte Interphase for Enhanced Anode Protection in Lithium Metal Batteries

**DOI:** 10.1021/acs.chemmater.5c01666

**Published:** 2025-10-20

**Authors:** Juhi Juhi, Mariana Vargas Ordaz, Sara Drvarič Talian, Elena Tchernychova, Wladyslaw Wieczorek, Janusz Lewiński, Robert Dominko

**Affiliations:** † Faculty of Chemistry, 201870 Warsaw University of Technology, Noakowskiego 3, 00-664 Warsaw, Poland; ‡ 68913 National Institute of Chemistry, Hajdrihova Ulica 19, 1000 Ljubljana, Slovenia; § ALISTORE - European Research Institute CNRS FR 3104,15 Rue Baudelocque, Amiens 80039, Cedex, France; ∥ Institute of Physical Chemistry, Polish Academy of Sciences, Kasprzaka 44/52, 01-224 Warsaw, Poland

## Abstract

Lithium metal has the potential to further increase the energy density of lithium batteries. However, its inherent instability with conventional liquid electrolytes, which leads to low coulombic efficiency, has limited its practical application. In this study, we introduce a simple, low-cost drop-casting method to create an artificial solid electrolyte interphase (SEI) on the lithium surface using cesium lead chloride (CsPbCl_3_). This inorganic protective coating enhances the interfacial stability between the lithium anode and the liquid electrolyte, effectively addressing common failure mechanisms. Symmetrical Li||Li cells with CsPbCl_3_–Li demonstrate cycling stability for 600 h at a current density of 1 mA/cm^2^ and a capacity of 1 mAh/cm^2^. When paired with LiFePO_4_ (LFP) cathodes (7.5 mg/cm^2^), CsPbCl_3_–Li||LFP batteries retained 99.46% capacity at 1C for 250 cycles, outperforming uncoated lithium anodes. The coating strategy provides a promising solution for producing stable lithium metal and paves the way for developing rechargeable batteries with high energy density.

## Graphical abstract

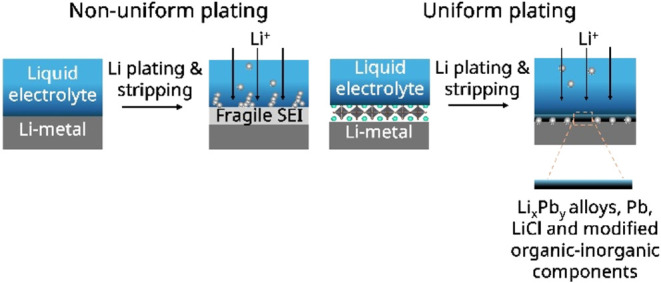

## 1 Introduction

The demand for high-energy-density batteries for energy storage and electric vehicles has intensified efforts to develop batteries that function effectively with lithium metal anodes (LMAs), due to their exceptionally high theoretical capacity (3860 mAh/g^1^), low density, and lowest reduction potential (−3.04 V vs standard hydrogen electrode).^1,2^ A LMA offers a 10-fold higher specific capacity than conventional graphite anodes used in state-of-the-art commercial lithium-ion batteries (LIBs), making it a highly promising candidate for next-generation battery technology.^3,4^ Despite these advantages, practical implementation remains challenging. Lithium (Li) metal exhibits poor thermodynamic stability with organic liquid electrolytes, leading to the formation of an unstable and fragile solid electrolyte interphase (SEI). This results in non–uniform Li deposition, high–surface–area Li (HSAL) formation, and continuous electrolyte consumption during cycling.^5^ Consequently, coulombic efficiency (CE) and cycle life are compromised, limiting widespread application and competitiveness.^6^ To address these issues and improve the cyclability of LMAs, numerous approaches have been developed, such as electrolyte engineering,^7–9^ 3D porous Li^+^ hosts,^10,11^ functional separators,^12,13^ and interface modifications.^14,15^

Among these, the application of *ex–situ* artificial solid electrolyte interphase (ASEI) to the lithium metal surface has emerged as one of the most effective approaches. These ASEIs regulate Li^+^ distribution and enhance the stability of the electrode/electrolyte interface, thereby mitigating dendrite growth and improving long–term battery performance.^16^ So far, a variety of coating materials, including inorganics,^17^ polymers,^18^ and hybrids^19^ have been applied to LMAs through various methods, such as solid gas reactions^20–22^ and wet chemical emulsion coating.^23–25^ The ideal ASEI should exhibit high Li^+^ conductivity, excellent electrolyte stability, electronic insulation, and mechanical flexibility to accommodate the volumetric changes of the LMA during cycling.^26^ However, achieving an optimal balance between structural stability and high ion conductivity remains a critical challenge, since most existing ASEI materials offer either mechanical robustness or high Li^+^ conductivity, but not both simultaneously.^17^ Hence, there is a constant search for new ASEI materials, and one of the emerging families of active materials in this field are metal halide perovskites (MHPs).

Hybrid perovskites and all–inorganic MHPs with the general formula ABX_3_, where “A” is a monovalent organic or inorganic cation ( e.g.,Cs^+^, Rb^+^ ), “B” is a divalent transition metal cation (generally Sn^2+^, Pb^2+^, etc.), and “X” is a halide anion^27,28^ have recently emerged as a promising class of materials for energy storage applications.^29–31^ The perovskite structure can accommodate extrinsic ions, and the diffusion of these harged ions can be effectively utilized for energy storage. Moreover, their chemical tunability and adjustable bandgap enable the formation of stable S/electrolyte interfaces.^32,17^ These properties make MHPs prospective candidates for ASEI, as they can effectively suppress dendritic growth and maintain interfacial integrity during cycling. By leveraging their electronic insulation and mechanical robustness, MHPs can address critical challenges associated with LMAs. Although both hybrid and inorganic MHPs are readily available via wet and solid–state synthesis,^33,34^ all–inorganic MHPs exhibit improved thermal and moisture stability compared to organic–inorganic hybrid perovskites, which suffer from volatile organic cations and environmental degradation.^35^ Consequently, greater focus has been devoted to exploring inorganic elements to replace organic cations, for instance, using cesium to form CsPbX_3_ all–inorganic perovskites.

In this report, we used cesium lead chloride perovskite (CsPbCl_3_) as a protective coating for LMAs by employing a simple drop–casting method to achieve uniform Li deposition and dendrite–free LMAs during cycling. Coated and uncoated Li–metal electrodes were evaluated in both symmetric (Li||Li) and asymmetric (Li||Cu) cell configurations to assess stability. Additionally, laboratory–scale Li–metal batteries (LMBs) with LFP cathodes were assembled as proof-of-concept cells to demonstrate the potential of CsPbCl_3_ coatings. To further understand the Li^+^ transport mechanism in the coating, X-ray photoelectron spectroscopy analysis of coated and uncoated electrodes was performed before and after cycling. Our results confirm that this protective layer effectively stabilizes the interface, promotes uniform Li^+^ deposition, and enhances long–term cycling performance, paving the way for the development of safer and more reliable LMBs.

## 2 Experimental Section

### 2.1 Materials

Cesium chloride (CsCl, Sigma–Aldrich, 99.9%), lead chloride (PbCl_2_, Sigma–Aldrich, 98%), sodium dodecyl sulfate (SDS, Sigma-Aldrich, 99.5%), lithium bis­(fluorosulfonyl)–imide (LiFSI, Solvionic, 99.9%), and lithium iron phosphate (LiFePO_4_/C, LFP, Targray, SLFP02002) were used as received. Tetrahydrofuran (THF, Honeywell, ≥99.9%) was dried using 4 Å molecular sieves for at least 5 days then refluxed overnight with a sodium–potassium (Na/K) alloy (approximately 1 mL/L) and purified by fractional distillation. The final water content of the distilled solvent was confirmed to be below 1 ppm by Karl Fischer titration, fluoroethylene carbonate (FEC, Alfa Aesar, 98%), anhydrous diethyl carbonate (DEC, Sigma-Aldrich, 99%), polyvinylidene fluoride (PVdF, Sigma-Aldrich), *N*-methyl-2-pyrrolidone (NMP, Merck, ≥99.7%), dimethyl sulfoxide (DMSO, Merck, ≥99.7%), and toluene (Merck, ≥99.5%) were used without further treatment.

### 2.2 Synthesis of CsPbCl_3_ Perovskite

CsPbCl_3_ perovskite was synthesized according to the previously reported method.^36^ Equimolar amounts of CsCl, PbCl_2_, and SDS powder were stirred in DMSO at room temperature for 1.5 h. After complete dissolution, the precursor solution was injected into toluene (as an antisolvent) under vigorous stirring, forming a white colloidal solution. After stirring for 2 min, the solution was centrifuged at 8000 rpm for 10 min. The supernatant was discarded, and the CsPbCl_3_ precipitate was dispersed in toluene. Two additional washing cycles were performed under the same centrifugation conditions. Finally, the white CsPbCl_3_ powder was dried overnight in a vacuum oven at 80 °C. The obtained perovskite was subsequently characterized.

### 2.3 Fabrication of CsPbCl_3_ Protected Li-Electrode

Before the coating process, lithium metal disks with a thickness of 500 μm were punched to a diameter of 12 mm and mechanically polished using a polystyrene weighing vessel. The coating dispersion was prepared by grinding a fixed amount (20 mg) of CsPbCl_3_ perovskite in a mortar and pestle for 5–8 min to obtain a fine powder. The ground perovskite was then dispersed in 1.5 mL of anhydrous tetrahydrofuran (THF), and the mixture was stirred vigorously for 24 h to achieve a homogeneous dispersion. All procedures were conducted in an inert atmosphere within an argon–filled glovebox (MBraun Unilab, with H_2_O and O_2_ levels <1 ppm). The protective coating was applied using a drop–casting technique, with 37.5 μL/cm^2^ of the dispersion deposited onto the polished lithium metal surface in four successive layers to achieve a total loading of 2 mg/cm^2^.

### 2.4 Battery Assembly and Electrochemical Testing

All test cells were assembled in an argon-filled glovebox (O_2_/H_2_O <1 ppm), and all electrochemical tests were performed in pouch cell format using a VMP3 Biologic potentiostat/galvanostat. For Li||Li symmetric cells with Cu contacts, two Li electrodes were stacked with a pressed Celgard 2320 separator (PP/PE/PP, 16 μm thickness) and an electrochemically active area of 0.502 cm^2^ between them to minimize edge effects.^37^ Galvanostatic stripping/plating tests were carried out on Li||Li symmetric cells incorporating either bare or coated Li electrodes. The tests were conducted at a constant current density of 1 mA/cm^2^ with a plating capacity of 1 mAh/cm^2^.

Coulombic efficiency (CE) measurements for Li||Cu cells were performed following a previously reported protocol,^38^ using both bare and coated Cu electrodes. The procedure began with a single preconditioning cycle involving lithium plating and stripping at a capacity of 4 mAh/cm^2^ and a current density of 0.4 mA/cm^2^. Subsequently, a lithium reservoir was formed by plating lithium onto the Cu electrode at the same current density and capacity. This was followed by 10 plating/stripping cycles with an areal capacity of 0.5 mAh/cm^2^, maintaining the current density at 0.4 mA/cm^2^. Finally, the remaining lithium was fully stripped from the Cu electrode at 0.4 mA/cm^2^, with the upper voltage limit set to 1 V. The CE was calculated by comparing the lithium capacity deposited during reservoir formation with the capacity recovered during the final stripping step. Additionally, a modified version of the protocol was employed, in which three preconditioning cycles were performed instead of one to study the impact of initial cycling on CE. For all experiments, 20 μL of 1 M LiFSI in FEC/DEC (1:2) electrolyte was used..

The full cell battery configuration was evaluated by coupling bare and coated Li anodes with LiFePO_4_ (LFP) cathodes with a mass loading of approximately 7.5 mg/cm^2^ of active material. LFP cathodes were prepared using a planetary ball mill equipped with a 12 mL stainless steel container and five stainless steel balls (16 mm in diameter). A mixture of LFP, conductive carbon black (C65), and polyvinylidene fluoride (PVdF) in a weight ratio of 90:5:5 was milled at 300 rpm for 30 min in the presence of *N*-methyl-2-pyrrolidone (NMP) as the solvent to obtain a homogeneous slurry. The resulting slurry was uniformly coated onto carbon-coated aluminum foil using a doctor blade set with a 200 μm gap. The coated electrodes were subsequently dried under vacuum at 100 °C overnight. After drying, the cathode films were punched into 10 mm diameter disks. The disks were then compressed at a pressure of 2 tons/cm^2^ to ensure uniformity. The prepared cathodes were stored in an argon-filled glovebox to prevent exposure to moisture and air. LMBs were tested in a potential range between 2.5 and 3.6 V vs Li^0^/Li^+^ with three formation cycles at C/10, followed by cycling at 1C, using 30 μL of 1 M LiFSI in FEC/DEC (1:2) electrolyte.

*Operando* electrochemical impedance spectroscopy (EIS) was utilized to evaluate the interfacial and diffusion resistance of both bare and coated Li electrodes during cell cycling. For these experiments, Li||Li symmetric cells were assembled and allowed to stabilize for 24 h while measuring EIS at open circuit voltage (OCV). The OCV EIS spectra were recorded from 1 MHz to 1 mHz, using a sinusoidal potential amplitude of 10 mV (root-mean-square, rms). *Operando* EIS was carried out under a current density of 1 mA/cm^2^ while maintaining an areal capacity of 1.0 mAh/cm^2^. The alternating current amplitude was 100 μA/cm^2^, and the frequency range was between 1 MHz and 20 mHz. Each cell was cycled for a total of 20 cycles under these standard cycling conditions.

### 2.5 Characterization

Scanning electron microscopy (SEM, Supra 35VP, Carl Zeiss), equipped with an energy-dispersive X-ray spectrometer (INCA Energy 400, Oxford Instruments, U.K.), was utilized to investigate the thickness, morphology, and microstructure of the coating. Sample preparation was performed in an argon-filled glovebox to prevent atmospheric exposure, and the samples were transferred to the SEM chamber using a custom-designed vacuum transfer holder. The holder was opened only under reduced pressure within the SEM chamber to maintain sample integrity.

X-ray photoelectron spectroscopy (XPS) was performed using a VersaProbe III AD (Physical Electronics, Chanhassen, USA) with a monochromated Al–Kα1 X-ray (1486.7 eV) excitation source. Spectra were acquired from a 1 mm^2^ area using a 200 μm spot size. The perovskite powder and electrodes were placed on nonconductive double-sided tape under “floating” conditions. High-resolution spectra were measured at 69 eV pass energy with 0.05 eV steps. Charge neutralization was employed, and the energy scale of the XPS spectra was calibrated by setting the C 1s peak of carbon to a binding energy of 284.8 eV. The spectra were analyzed using UIVAC-PHI Multipack software, with a binding energy error of ±0.65 eV for all peak fits. A smart background was applied to all spectra.

X-ray diffractogram (XRD) was acquired on an X′Pert PRO diffractometer (Malvern Panalytical) with Cu Kα radiation (λ = 1.541874 Å). The powder sample was prepared on a zero-background Si holder and measured in the 2θ range from 5 to 80° with a step size of 0.008° per 800 s using a fully opened Pixcel detector.

## 3 Results and Discussion

### 3.1 Optimization of CsPbCl_3_ Protective Coating on Li–Metal

The structural, elemental, and morphological optimization of the synthesized CsPbCl_3_ perovskite powder was performed using XRD, XPS, and SEM, with the corresponding results shown in Figures S1, S2, and S3. The XRD pattern of the CsPbCl_3_ sample displayed sharp, well-defined peaks, indicating high crystallinity. Detailed analysis of the diffraction pattern confirmed the formation of a pure tetragonal perovskite phase with no detectable secondary phases or impurities. The crystal structure and phase purity were further validated by full-profile Rietveld refinement, performed using the *P*4*mm* space group (Figure S1). To determine the chemical composition of the CsPbCl_3_ powder, XPS was performed. The survey and high-resolution XPS spectra of the characteristic cesium (Cs), chlorine (Cl,) and lead (Pb) peaks are presented in Figure S2. XPS analysis (Figure S2) revealed the presence of all expected constituent elements, including Cs, Pb, Cl, sodium (Na), oxygen (O), and residual sulfur (S). The high-resolution spectra displayed characteristic binding energies for Cs, Pb, and Cl that are in good agreement with previously reported values for CsPbCl_3_ perovskite,^39,36^ supporting the elemental integrity of the material. SEM imaging combined with EDX (Figure S3) showed well-defined cubic-shaped CsPbCl_3_ crystals with an average particle size of approximately 170 nm.

To determine the optimal CsPbCl_3_ perovskite loading for surface coatings, a galvanostatic cycling test was conducted using Li||Li symmetric cells, as shown in Figure S4. Coatings with three different areal loadings, 1, 2, and 4 mg/cm^2^, were evaluated. Among these, the 2 mg/cm^2^ loading exhibited the lowest overpotentials, which were maintained for 200 h of extended cycling compared to the other two loadings. Based on these results, a CsPbCl_3_ loading of 2 mg/cm^2^ was selected for subsequent electrochemical testing. The morphology of pristine and CsPbCl_3_–coated Li was analyzed by SEM (Figure 1). The surface image of the coated Li–metal shows a uniform distribution of CsPbCl_3_ cubes across the Li–metal surface (Figure 1 b,c). The thickness of the protective layer was determined to be approximately 5 μm (Figure 1d). Moreover, EDX mapping (Figure 1e–g) confirmed the homogeneous distribution of Cl, Cs, and Pb elements within the CsPbCl_3_ coating on the Li-metal surface.

**Figure 1:**
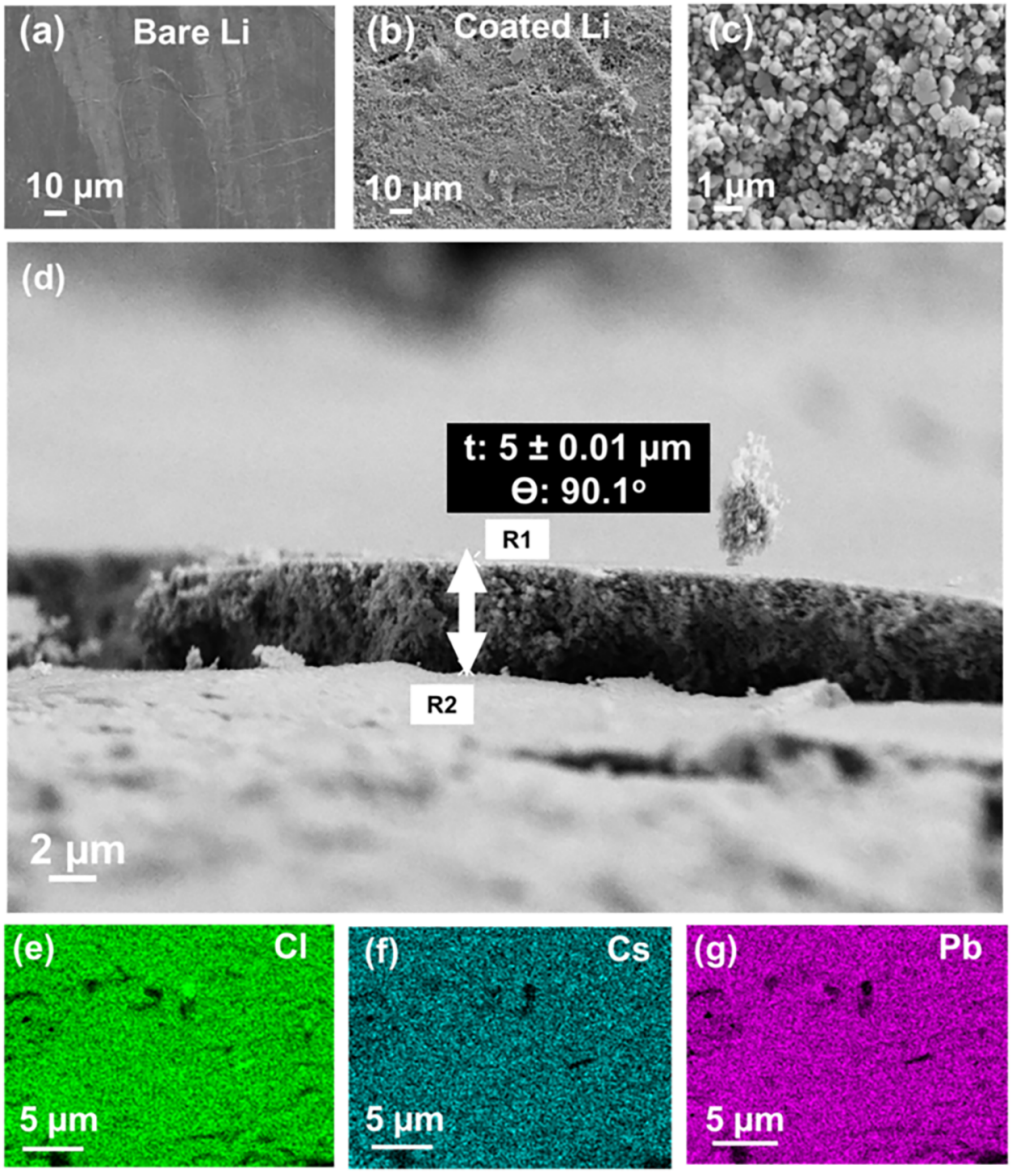
SEM images of (a) bare Li–metal, (b) and (c) coated Li–metal with 2 mg/cm^2^ CsPbCl_3_, (d) cross–section of coating applied on Li–metal. EDX mapping of coated Li showing the elemental distribution of (e) Cl, (f) Cs, and (g) Pb.

To better understand the chemical composition and chemical state of each element in the perovskite coating on Li– metal, XPS measurements were conducted. The survey XPS spectrum is shown in Figure S5. The coating chemistry is identical to that of powder CsPbCl_3_, with Cs, Pb, Cl, S, Na, and O present. The S, Na, and O peaks originate from SDS, which was used during CsPbCl_3_ synthesis. The XPS binding energies (BEs) of the characteristic Cs, Pb, and Cl peaks for coated–Li are presented in Figure 2. Two peaks centered at 738.2 and 724.3 eV correspond to the 3d_3/2_ and 3d_5/2_ bands of Cs, respectively. The Pb 4f_5/2_ and Pb 4f_7/2_ peaks are located at 143.1 and 138.2 eV. The Cl 2p_3/2_ and Cl 2p_1/3_ peaks are centered at 199.5 and 197.8 eV, respectively. These BEs are consistent with those of pristine CsPbCl_3_ powder (Figure S2), indicating that no significant chemical changes occurred during the coating process.

**Figure 2:**
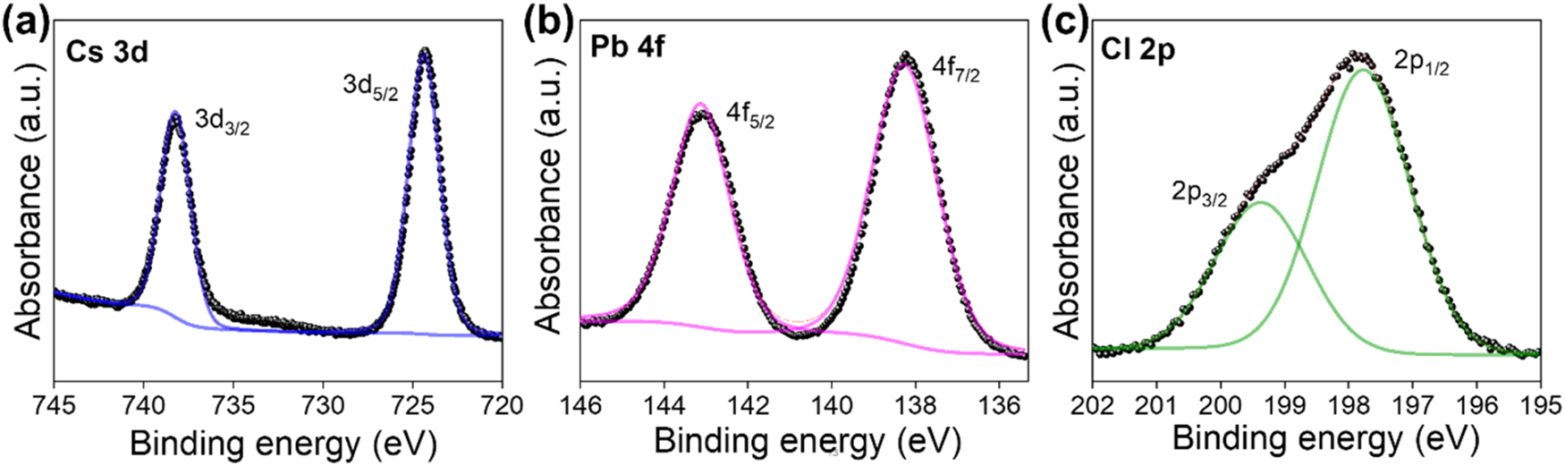
XPS spectra of the CsPbCl_3_–coated Li (a) Cs 3d, (b) Pb 4f, and (c) Cl 2p peak positions.

### 3.2 Electrochemical Characterization of Li||Li Symmetric Cells

To evaluate the electrochemical performance of Li–metal electrodes and assess the protective effect of CsPbCl_3_ coatings in stabilizing the LMA, Li||Li symmetric cells were assembled and tested. The ionic conductivity of the electrolyte was determined to be 4 mS/cm at 25 °C by comparing the resistance intercept values to those of cells with LP40 electrolyte, which has a known conductivity of 8 mS/cm at the same temperature. The transference numbers for both bare and coated Li cells were determined using different methods (Figure S6). The values for both cells were similar, 0.10 ± 0.03 and 0.09 ± 0.02, respectively, supporting the hypothesis that the coating is porous and conduction occurs through it in the electrolyte filling the pores.

The electrodes then underwent continuous plating and stripping with an areal capacity of 1 mAh/cm^2^ and a current density of 1 mA/cm^2^ per charge/discharge cycle. This approach enabled monitoring of overpotential variations during cycling, providing insights into mass transport phenomena at the electrolyte/Li–metal interface and facilitating evaluation of the coating stability.^40^ The cell with bare Li–metal exhibited an initial overpotential of 140 mV (Figure 3b, panel 1), while the coated Li cell showed an overpotential of 60 mV. This difference in overpotential is influenced by the initial interfacial resistance, as confirmed by EIS measurements at open circuit voltage (OCV) presented in Figure S7. The EIS results indicate that the total resistance (R_total_) of the bare Li electrode is significantly higher (∼ 217 Ω), resulting in a larger initial overpotential (∼ 109 mV). In contrast, coated Li exhibited lower impedance (∼ 115 Ω), corresponding to ∼ 58 mV of overpotential. As shown in Figure S7, most of the difference in total resistance arises from the change in SEI resistance (174 Ω for bare vs 35 Ω for coated), while diffusional resistance remains similar (50 Ω bare vs 65 Ω coated). This suggests that the reduced overpotential observed can be attributed to the lower interfacial resistance provided by the coating.

**Figure 3:**
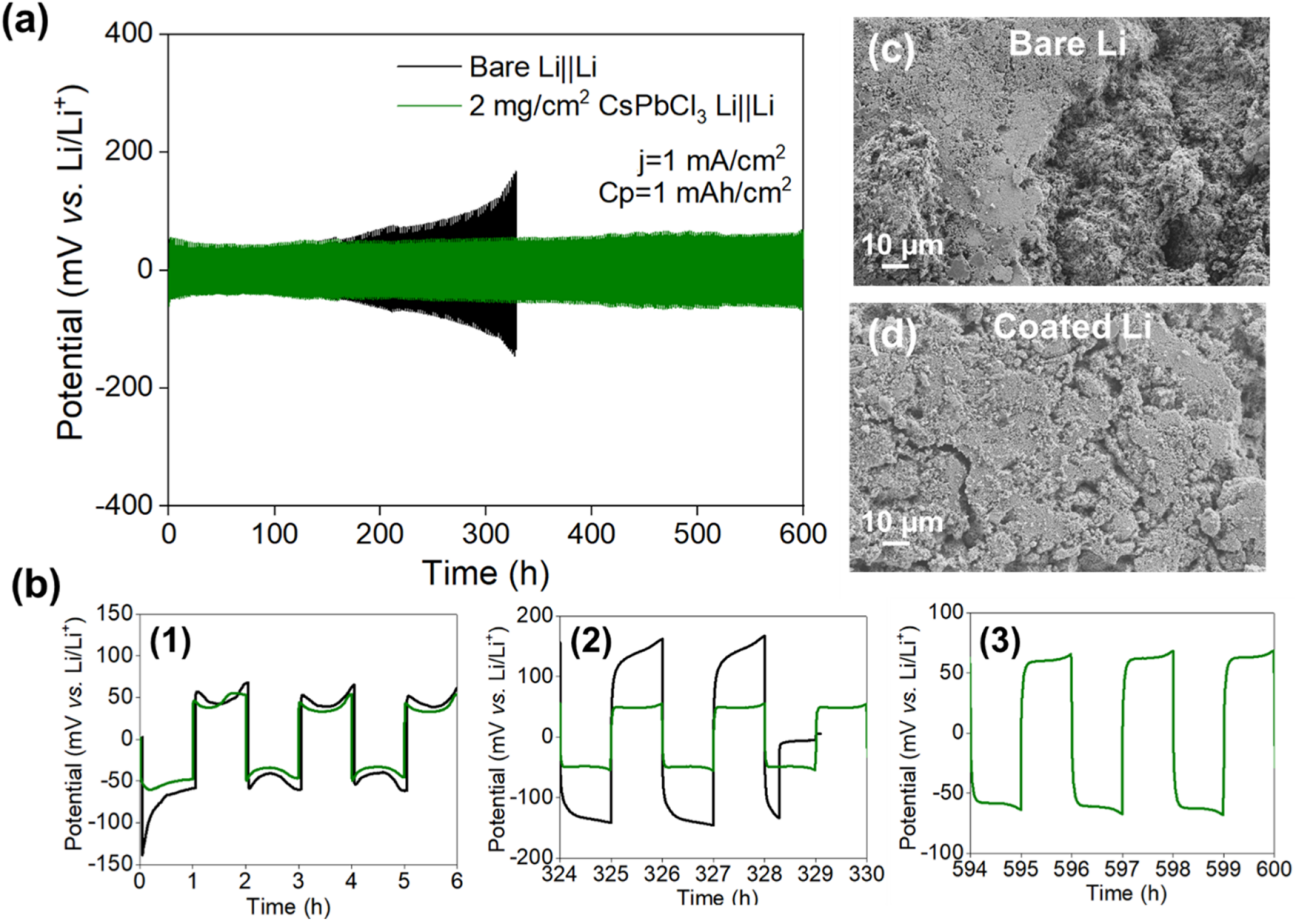
(a) Galvanostatic cycling of bare and coated Li||Li symmetric cells at an areal capacity of 1 mAh/cm^2^ with a current density of 1 mA/cm^2^ in 1 M LiFSI in FEC/DEC (1:2), and (b) represents the detailed voltage profiles. SEM micrographs of (c) bare Li–metal and (d) CsPbCl_3_–coated Li–metal after 20 cycles.

Significant differences emerged between the bare Li||Li cells and the coated electrodes during extended cycling. The bare Li||Li symmetric cells exhibited a noticeable increase in overpotential after 170 h of cycling, ultimately short–circuiting after 327 h due to the formation of HSAL. In contrast, the CsPbCl_3_–coated Li||Li cells extended the cycle life of the Li–metal to 600 h, with only a small increase in overpotential to 80 mV. The extended cycling life and stable performance of the coated Li–metal electrodes demonstrate the protective layer’s ability to improve cell stability. Moreover, the cycling stability of the coating in Li||Li symmetric cells was also confirmed under higher current densities of 2 and 4 mA/cm^2^. The Li||Li symmetric cell tests show that the CsPbCl_3_ coating improves the electrochemical stability of Li–metal electrodes compared to bare Li. At a current density of 2 mA/cm^2^ and an areal capacity of 2 mAh/cm^2^ (Figure S8a), the bare Li||Li cell shows a progressive increase in overpotential with pronounced fluctuations over time, indicating unstable Li plating/stripping behavior. In contrast, the CsPbCl_3_–coated Li||Li cell exhibits a relatively lower overpotential for nearly 200 h, indicating the coating’s ability to regulate Li^+^ deposition. At 4 mA/cm^2^ and an areal capacity of 4 mAh/cm^2^ (Figure S8b), the bare Li||Li cell fails within approximately 72 h. Meanwhile, the CsPbCl_3_–coated Li||Li cell continues to operate for about 100 h; however, a gradual increase in overpotential is observed under these harsher conditions. These results demonstrate that the CsPbCl_3_ coating acts as a protective interphase that can promote uniform Li^+^ deposition. However, at higher current densities and capacities, the protection is less effective, suggesting that further optimization of the coating is required. To further investigate the impact of the coating, SEM analysis was performed on the electrode surfaces after 20 cycles. The bare lLi electrode displayed a typical porous, high–surface–area structure with significant quantities of mossy Li covering most of the electrode surface (Figure 3c). In contrast, the coated electrode surface retained a morphology close to the pristine coating (Figure 1), suggesting the coating remained nearly intact (Figure 3d).

To investigate the factors contributing to the improved cycling stability of coated Li–metal electrodes, we performed *operando* EIS^41^ on symmetric Li||Li cells with both bare and coated electrodes (see Note 2 in the SI for further details). Figure 4 shows the impedance spectra as stacked Nyquist plots rotated by 90°, with the low–frequency data points precisely aligned with the simultaneously measured overpotential values (shown in red and green). Arc assignment was done according to a previously published transmission line model for pristine Li and Li with high surface area deposits.^42^ The resistance and capacitance of the high–frequency arc measured in both coated and bare Li EIS spectra corresponded well with the model’s contribution from Li^+^ migration in the compact SEI layer. The reported resistance values were extracted directly from the EIS spectra as the difference in resistance between the beginning and end of the impedance arc, with no fitting performed.

**Figure 4:**
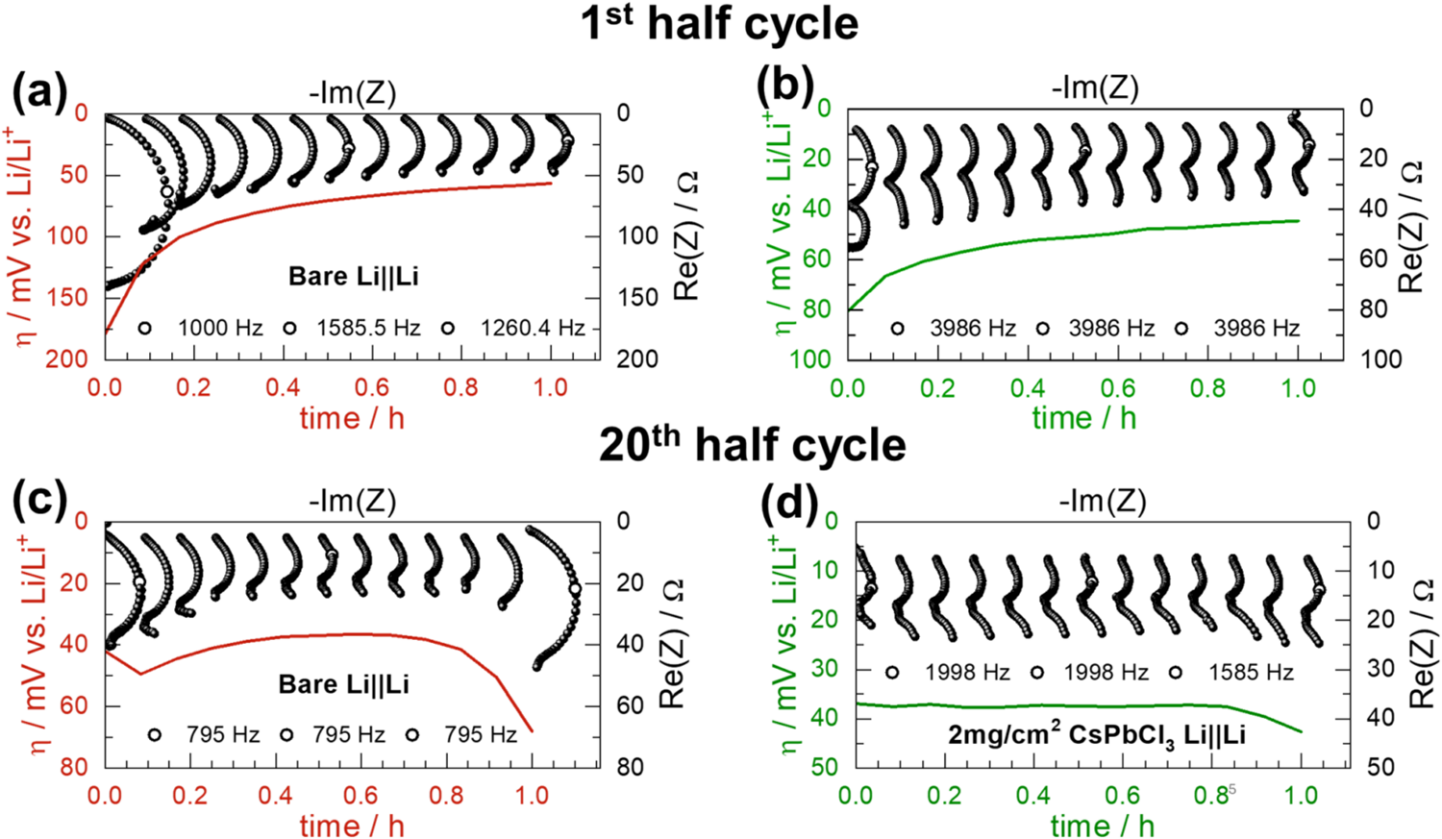
*Operando* impedance measurement (black points, scale on top and right) and overpotential (red and green curve, scale on bottom and left). Symbols mark peak frequencies of the 1st, 7th, and 13th spectra. First half cycle: (a) bare Li||Li and (b) CsPbCl_3_–coated Li||Li. 20th half cycle: (c) bare Li||Li and (d) CsPbCl_3_–coated Li||Li.

The coated Li electrode (Figure 4b) exhibits a lower initial interfacial resistance (*R*_SEI_) of approximately 32 Ω in the high–frequency region, compared to about 137 Ω for the bare Li electrode (Figure 4a). Furthermore, *R*_SEI_ remains relatively stable throughout the first half cycle for the coated electrode, with a final *R*_SEI_ value of ∼ m20 Ω, corresponding to a 37.5% reduction. In contrast, the bare Li shows significant variation in the arc size over time, with the final *R*_SEI_ of ∼ 40 Ω, representing a 71% decrease from its initial value. This indicates substantial HSAL formation, which contributes to increased interfacial heterogeneity and instability during cycling.

These differences become even more pronounced over extended cycling. By the 20th cycle, *operando* EIS data reveal a substantial increase in overpotential during the second half of the stripping cycle for the bare Li electrode (Figure 4c), whereas the coated electrode maintains a more stable overpotential profile (Figure 4d). The rise in overpotential for bare Li is attributed to the progressive consumption of accessible HSAL deposits. Once these deposits are depleted, Li stripping must proceed from the bulk of the flat electrode, leading to pitting and a plateau in overpotential, as reported in the literature.^43^

Overall, *operando* EIS analysis highlights critical differences in interphase evolution between bare and CsPbCl_3_–coated electrodes. In bare Li||Li cells, *R*_SEI_ exhibited substantial fluctuations of up to ∼ 40%, corresponding to ongoing HSAL formation and SEI growth.^34^ In contrast, the coated electrodes showed improved interfacial stability, with *R*_SEI_ variations limited to ∼ 10%. These findings support the role of the CsPbCl_3_ coating in suppressing HSAL formation, maintaining a stable electrode/electrolyte interface, and enhancing the cycling performance of Li–metal electrodes.

*Ex–situ* XPS analysis (after the first and 20th cycles) was performed to understand the critical insights into the chemical evolution of the CsPbCl_3_ coating in the bulk during cycling and its role in the enhanced interfacial stability observed in *operando* impedance results. Figure 5a presents the Pb 4f XPS spectra of CsPbCl_3_–coated Li electrodes before and after cycling. After the first and 20th cycles, the characteristic Pb^2+^ doublet associated with CsPbCl_3_ exhibits a slight shift toward lower BE, suggesting partial reduction of Pb^2+^ species. Additionally, new peaks emerge at even lower BE values (∼ 141 eV and ∼ 136 eV), corresponding to metallic Pb^0^. This indicates the formation of Li_*x*_Pb_*y*_ alloys and Pb at the electrode surface during cycling. These Li_*x*_Pb_*y*_ alloys serve as lithiophilic nucleation sites, lowering the energy barrier for Li^+^ deposition.^44^ This observation aligns with the galvanostatic cycling results (Figure 3a) and SEM images (Figure 3d), where the coated electrode maintains a relatively smooth surface morphology postcycling, in contrast to the porous, mossy Li growth seen on the bare Li electrode (Figure 3c).

**Figure 5:**
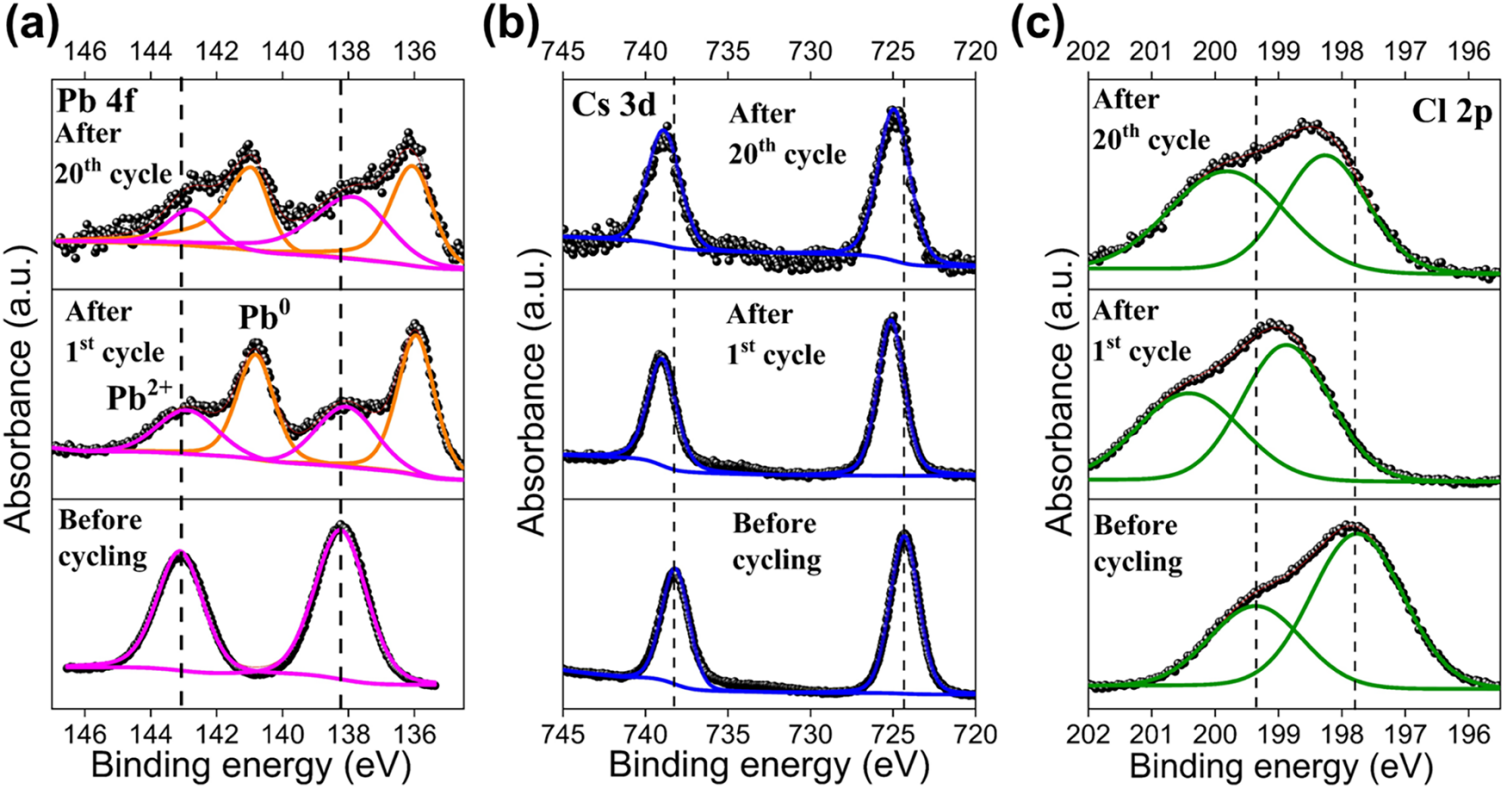
XPS spectra of the CsPbCl_3_–coated Li before and after cycling (a) Pb 4f, (b) Cs 3d, and (c) Cl 2p peak positions.

A minor shift (∼ 0.5 eV) of the Cs 3d (Figure 5b) peaks toward higher BE was also observed, but this is within the instrumental energy resolution (∼ 0.6 eV) and is considered negligible. In contrast, the Cl 2p peaks (Figure 5c) exhibited an unexpected shift toward higher BE after the first cycle, contrary to the typical lower BE expected for LiCl. This shift suggests significant interfacial interactions between decomposition products and the organic components of the electrolyte (FEC: DEC). As shown in Figure 5c, the higher BE of Cl after the first cycle could result from the coordination of LiCl with polar functional groups such as −CO_3_ and −O– within the SEI matrix, modifying their electronic environments. After 20 cycles, the Cl 2p peaks shift slightly back toward lower BE, though they remain higher than coated Li before cycling. This may suggest a growing contribution of inorganic LiCl near the surface, likely due to continued perovskite decomposition and the release of Cl^–^ ions, which then form LiCl domains. Similarly, the Li 1s spectra (Figure S9) show a slight shift (∼ 0.7 eV) toward higher BE, which could be attributed to the coordination of Li^+^ with Cl^–^ or organic species such as Li_2_CO_3_ or ROLi. The F 1s spectra (Figure S10) consistently display two peaks, LiF (∼ 684.5 eV) and organic fluorides (∼ 688.4 eV) on both bare and coated electrodes after 1 and 20 cycles. The O 1s spectra (Figure S11) also reveal two dominant peaks at 531.5 and 533.6 eV, corresponding to carbonyl (–C=O) and alkoxy (−C–O) groups, respectively.

Collectively, these findings suggest that the CsPbCl_3_ coating undergoes a conversion-type reaction upon cycling (schematic below), leading to the formation of a hybrid SEI composed of Li_x_Pb_y_ alloys, Pb, LiCl, and modified organic/inorganic species. This composite SEI offers enhanced ionic conductivity and mechanical robustness, contributing to reduced interfacial resistance and improved cycling stability. However, a full discussion on the SEI evolution requires *operando* XRD and in-depth XPS analysis, which is beyond the scope of this work and will be addressed in a follow-up study.

1 $$\mathrm{CsPb}{\mathrm{Cl}}_{3}\to \mathrm{LiCl}+{\mathrm{Li}}_{x}{\mathrm{Pb}}_{y}+\mathrm{Pb}$$

### 3.3 Electrochemical Characterization of Li||Cu Asymmetric Cells

In symmetric Li||Li cells, the unlimited lithium reservoir hinders the accurate determination of CE. To address this limitation, asymmetric Li||Cu cells with a finite lithium supply were employed. In this setup, a fixed amount of Li (17.2 μm, corresponding to 4 mAh/cm^2^) was initially deposited onto the copper substrate. The cells were then cycled for 10 cycles at 0.5 mAh/cm^2^ per cycle (equivalent to 2.16 μm of lithium plated and stripped per cycle), followed by an exhaustive final stripping of Cu at 4 mAh/cm^2^. This method enables for precise CE calculation by comparing the total charge from the initial Li reservoir with the charge recovered during the final stripping process.^40^ Additionally, a high–capacity single preconditioning cycle was implemented to enhance the copper substrate’s stability, reducing passivation effects and mitigating the “ramp-up” phenomenon that can otherwise introduce inaccuracies in CE measurement.^40^ Figure 6 compares the performance of asymmetric Li||Cu cells with bare and coated copper substrates. Initially, the CE of the coated Cu (97.12%) was slightly lower than that of the bare Cu (97.5%), attributed to initial Li consumption due to the conversion reaction involving the CsPbCl_3_ coating.^45,46^ To offset this initial Li loss, the number of preconditioning cycles was increased from one to three. Interestingly, while the CE of the bare Cu decreased from 97.5 to 96.1% likely due to HASL formation, the CE of the coated Cu improved from 97.12% to 98%. This enhancement is presumably due to the complete conversion of CsPbCl_3_ into Li_*x*_Pb_*y*_ alloys, Pb, LiCl, and modified organic/inorganic interphase components (as discussed in the previous section), which promote improved Li utilization. Furthermore, similar to the behavior observed in symmetric cells, a smoother galvanostatic profile with improved kinetics was observed for the coated Cu consistent with the presence of Li_*x*_Pb_*y*_ alloys. These results indicate that the CsPbCl_3_ coating contributes to improved CE, thereby enhancing the practical feasibility of LMBapplications.

**Figure 6:**
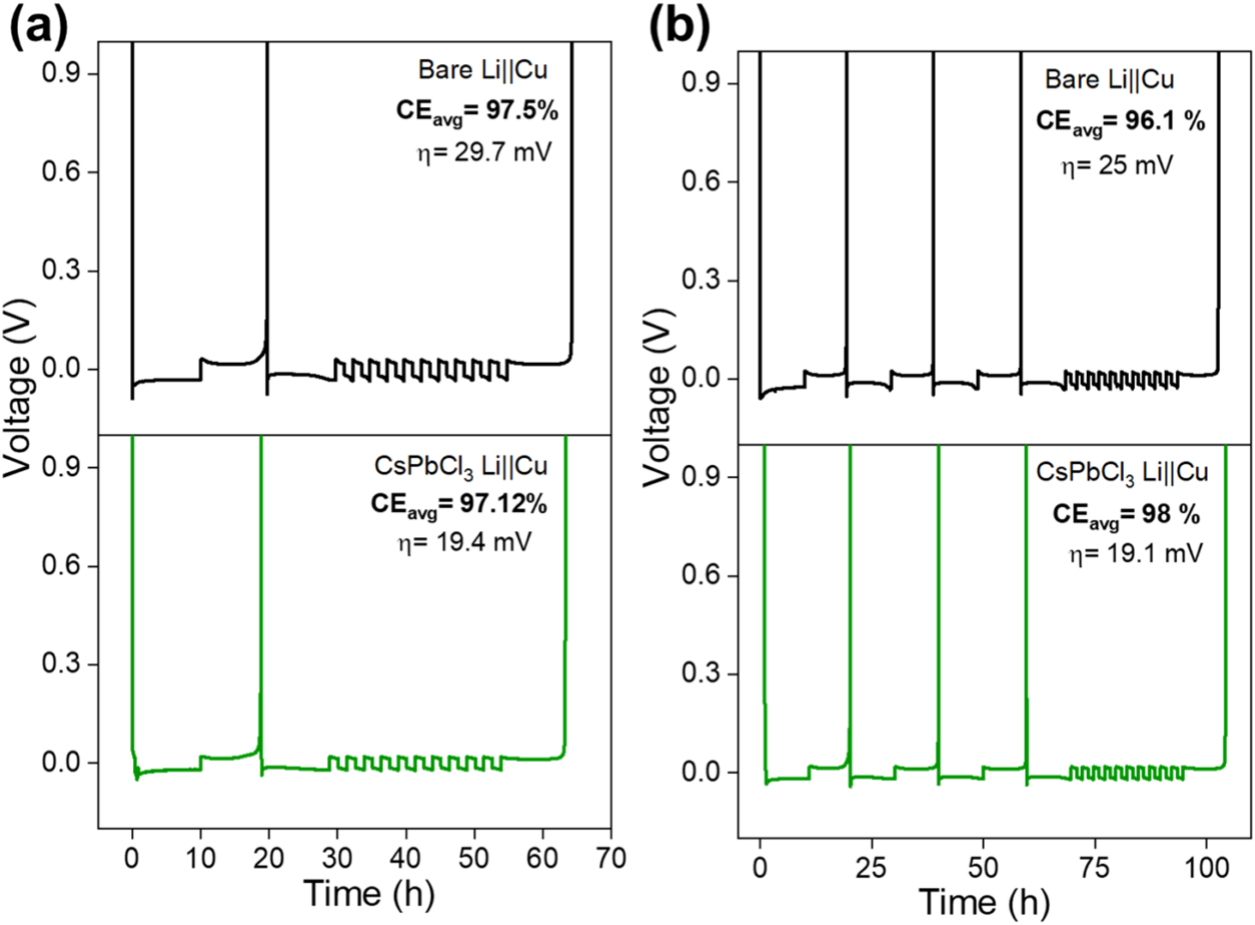
Utilization tests using Adams protocol to calculate CE of bare and CsPbCl_3_–coated Li||Cu cells: (a) one preconditioning cycle and (b) three preconditioning cycles.

### 3.4 Electrochemical Characterization of Li||LFP Full Cells

To evaluate the effectiveness of the CsPbCl_3_ coating, Li||LFP batteries were assembled by pairing bare and CsPbCl_3_–coated LMAs with LFP cathodes. The LFP electrodes had active material loadings of 7.6 and 7.5 mg/cm^2^, corresponding to areal capacities of 1.29 and 1.27 mAh/cm^2^, respectively. The cells underwent three initial formation cycles at C/10 before continuous cycling at a 1C rate. During the formation cycles, specific capacities of 145 and 156 mAh/g were achieved for the cells with bare and CsPbCl_3_–coated Li anodes, corresponding to 85.2 and 91.7% of the theoretical capacity of LFP (170 mAh/g), respectively. Under 1C cycling conditions, the bare Li cell exhibited a specific capacity of approximately 130 mAh/g with notable fluctuations. However, after 150 cycles, the reversible capacity retention dropped sharply to 91.19%, indicating degradation of the Li–metal anode. In contrast, the cell with the CsPbCl_3_–coated Li anode demonstrated a stable specific capacity of around 136 mAh/g over 250 cycles (Figure 7a), keeping a stable reversible capacity retention of 99.46%. Moreover, the CsPbCl_3_–coated Li anode battery also shows improved performance compared to the bare Li cell. As the current density increases, the CsPbCl_3_–coated Li||LFP can still deliver high discharge capacities of 163, 161.2, 156.5, 150.2, and 140.3 mAh/g at 0.1, 0.2, 0.5, 1, and 2C, respectively (Figure 7b). However, the cell with a bare Li anode displays inferior rate performance; the discharge specific capacities are 160.7, 159.8, 151.55, 135, and 109.5 mAh/g at 0.1, 0.2, 0.5, 1, and 2C, respectively. These results highlight the stabilizing effect of the CsPbCl_3_ coating.

**Figure 7:**
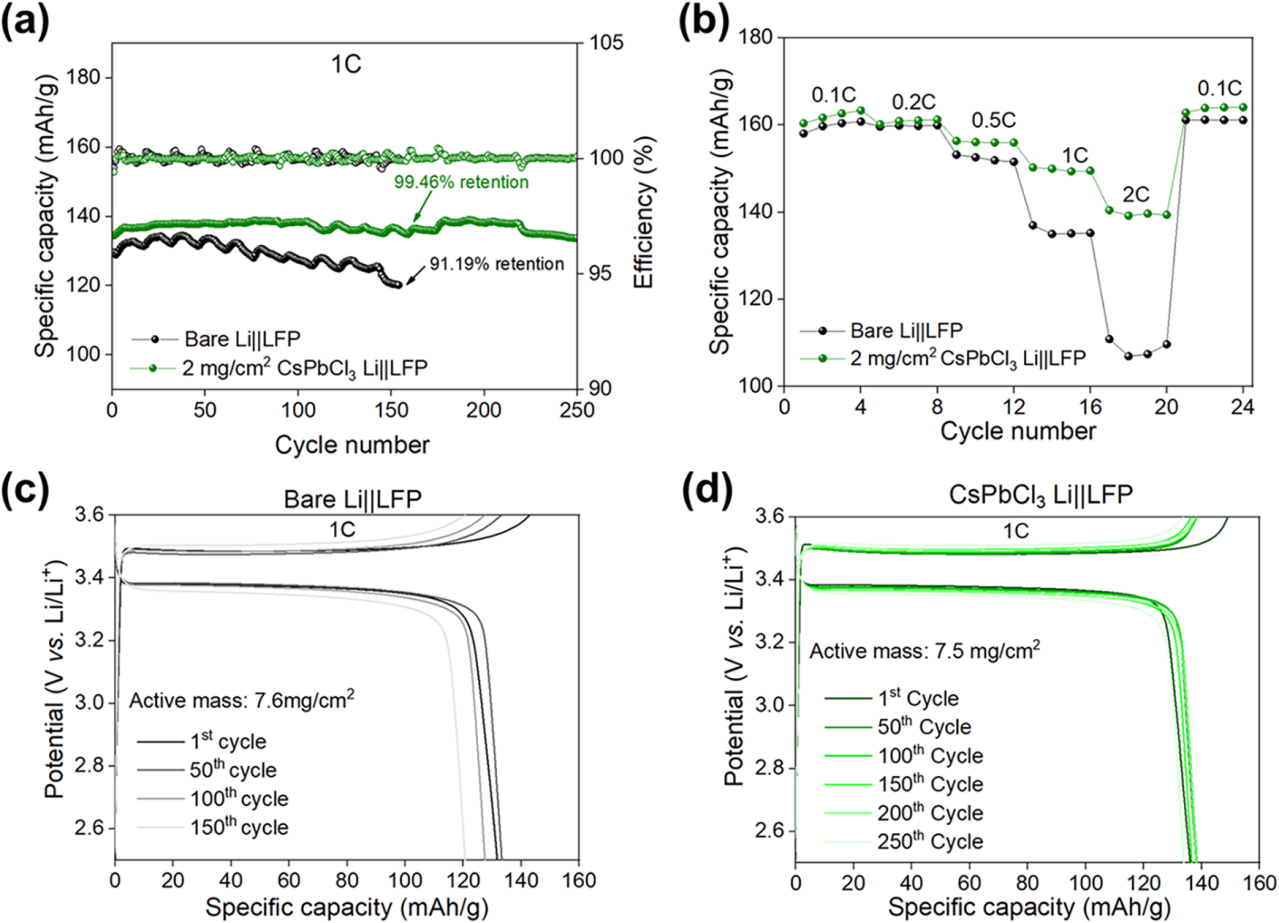
(a) Related capacity retention and CE during prolonged cycling, up to 250 cycles at 1C. (b) Rate capability of Li||LFP cells with (b) bare Li and (c) CsPbCl_3_–coated Li anodes at different rates from 0.1 to 2C. (c) Galvanostatic charge/discharge profiles of Li||LFP battery cells with bare Li anodes, at 1C, and (d) CsPbCl_3_–coated Li anodes.

## 4 Conclusion

In this work, we demonstrated that CsPbCl_3_ perovskite enables the formation of a stable Li–metal protective coating that extends cycle life and mitigates HSAL formation. The coating’s performance was tested in both symmetric Li||Li and asymmetric Li||Cu cells to evaluate its stability and CE, respectively. CsPbCl_3_–coated Li||Li cells exhibited longer and more stable cycle life, with a slower increase in overpotential compared to bare Li at a current density of 1 mA/cm^2^ and a capacity of 1 mAh/cm^2^. Using the modified Adam’s protocol, coated Li||Cu cells achieved an improved CE of 98%, while bare Li cells showed a lower CE of 96.1%. *Operando* EIS indicated that the CsPbCl_3_ coating suppresses HSAL formation and maintains a stable electrode/electrolyte interface. XPS results revealed that the CsPbCl_3_ coating reacts with Li–metal, forming a hybrid SEI composed of Li_*x*_Pb_*y*_ alloys, Pb, LiCl, and modified organic/inorganic components, which reduces interfacial resistance and improves cycling stability. Additionally, laboratory–scale full cells were assembled using a commercially relevant LFP cathode. CsPbCl_3_–coated Li anodes showed improved performance, with 99.46% capacity retention after 250 cycles, compared to 91.19% retention after 150 cycles for bare Li. These results confirm the positive effect of CsPbCl_3_ coatings in improving the long-term performance of LMBs.

## Supplementary Material



## Notes

Contains characterization of CsPbCl_3_ by XRD, XPS, and SEM; additionally, we show the optimization of CsPbCl_3_ perovskite loading for coating formulation, a detailed explanation of the Li–transference number study, and the setup for *operando* EIS study; our proposed technology is benchmarked with selected approaches from the literature (PDF)
