# Supplementary material for: Cesium Lead Chloride as an Artificial Solid Electrolyte Interphase for Enhanced Anode Protection in Lithium Metal Batteries
Source: Chem Mater. 2025 Oct 20;37(21):8745–54. doi: 10.1021/acs.chemmater.5c01666 (PMC12613310; doi:10.1021/acs.chemmater.5c01666)
Supplement: Supplementary file 1 [file cm5c01666_si_001.pdf]

Supporting information

Cesium Lead Chloride as Artificial Solid Electrolyte Interface for Enhanced Anode Protection in Lithium Metal Batteries

Juhi Juhi[a,b], Mariana Vargas Ordaz[b], Sara Drvarič Talian[b], Elena Tchernychova[b], Wladyslaw Wieczorek*[a], Janusz Lewiński*[a,d], Robert Dominko*[b,c],

[a] *Faculty of Chemistry, Warsaw University of Technology, Noakowskiego 3, 00-664 Warsaw, Poland*

[b] *National Institute of Chemistry, Hajdrihova Ulica 19, 1000 Ljubljana, Slovenia*

[c] *ALISTORE - European Research Institute, CNRS FR 3104, 15 Rue Baudelocque, Amiens 80039 Cedex, France*

[d] Institute of Physical Chemistry, Polish Academy of Sciences, Kasprzaka 44/52, 01-224 Warsaw, Poland.

* Emails: wladyslaw.wieczorek@pw.edu.pl; janusz.lewinski@pw.edu.pl; robert.dominko@ki.si;

1. **Characterization of $CsPbCl_3$ perovskite**

1.1. XRD analysis

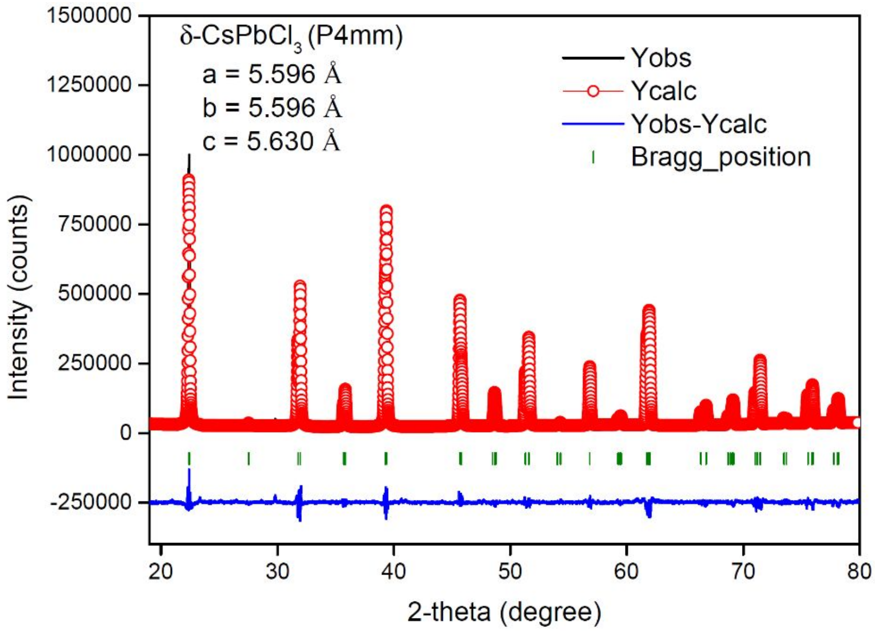

Figure S1. XRD pattern (solid black line, Yobs) for $CsPbCl_3$ powder. Refinement analysis (red dotted line, Ycalc) by the Rietveld method using the profile fitting FullProf Suite program. The horizontal (blue solid line) represents the residual, and the vertical lines (green solid lines) are the Bragg positions.

1.2. XPS analysis

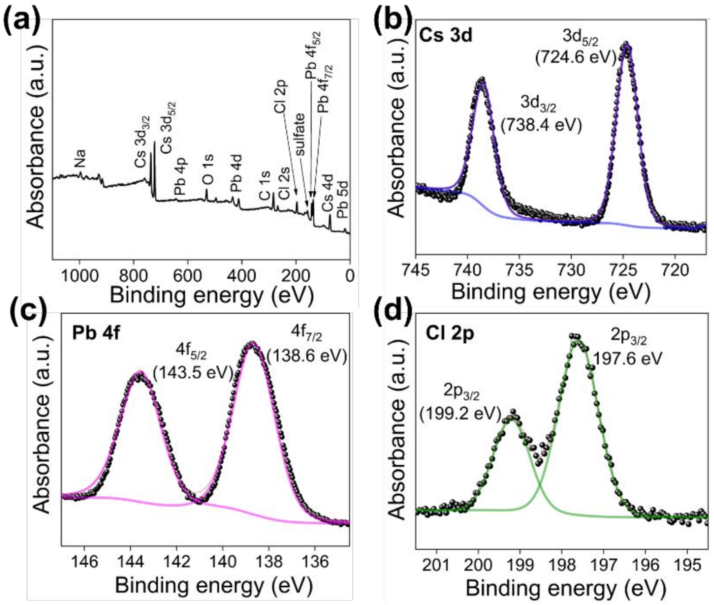

Figure S2. (a) Survey XPS spectrum and high-resolution XPS spectra (b) Cs 3d, (c) Pb 4f, and (d) Cl 2p peak positions of $CsPbCl_3$ powder.

1.3. SEM analysis

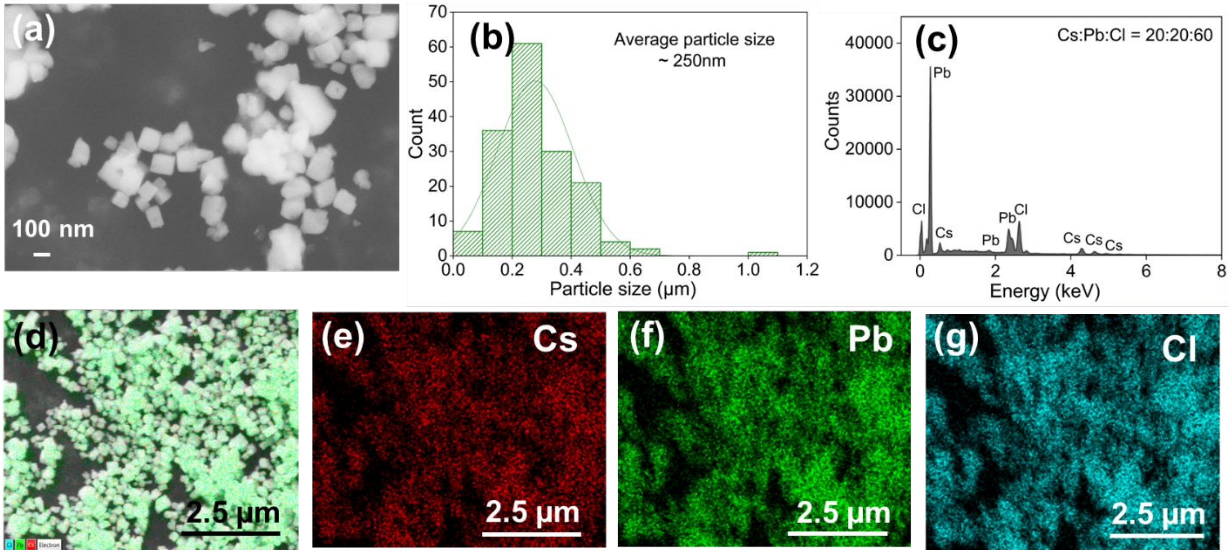

Figure S3. (a) SEM images of $CsPbCl_3$ powder, (b) average particle size, (c) EDX spectra of $CsPbCl_3$ powder. (d) EDX mapping of $CsPbCl_3$ powder showing the elemental distribution of (e) Cs, (f) Pb, and (g) Cl

2. Optimization of $CsPbCl_3$ perovskite loading for coating formulation

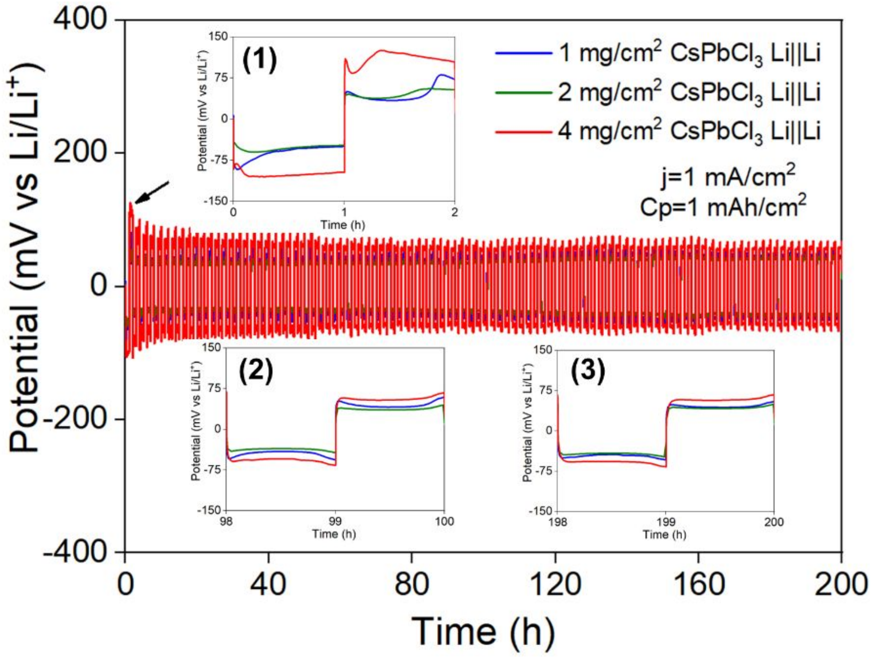

Figure S4. Optimization of CsPbCl3 perovskite content evaluated by galvanostatic cycling in

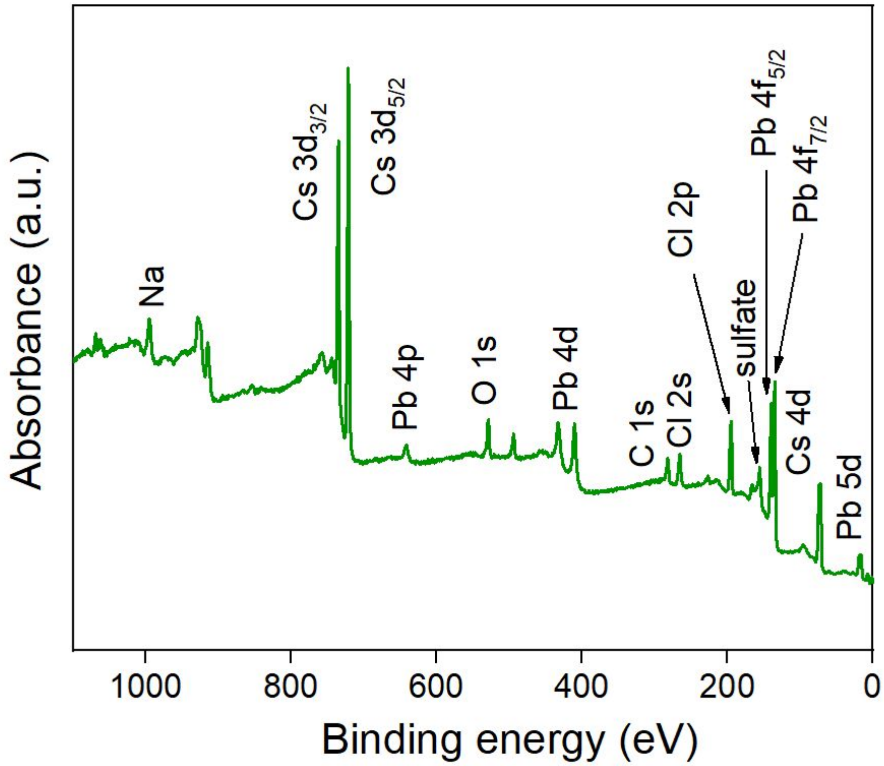

Figure S5. Survey XPS spectrum of $CsPbCl_3$-coated Li electrode.

3. Li||Li symmetric cells

Note 1: Li-transference number study

Three different approaches were used to determine the transference number for both bare Li and coated Li cells: the Bruce-Vincent polarisation method, low-frequency EIS measurement of pristine cells at OCV, and first-spectrum operando EIS measurement. For each method, the electrolyte resistance (R_{el}) and Warburg resistance (RW) were obtained. The electrolyte resistance was determined as the high-frequency resistive intercept from the impedance spectra. The Warburg resistance was determined as the low-frequency resistive intercept in the PEIS@OCV measurements (Fig. S6b, e) or calculated as the difference between the sum of the electrolyte and SEI resistances and the total resistance calculated from the overpotential and applied current (Fig. S6a, c, d, f). These values were then used to calculate the transference number using equation (1).:

$$t = \frac{R_{el}}{R_{el}+R_W} \quad (1)$$

For bare Li cells, the transference number was 0.10 ± 0.03 and for the coated Li cell 0.09 ± 0.02.

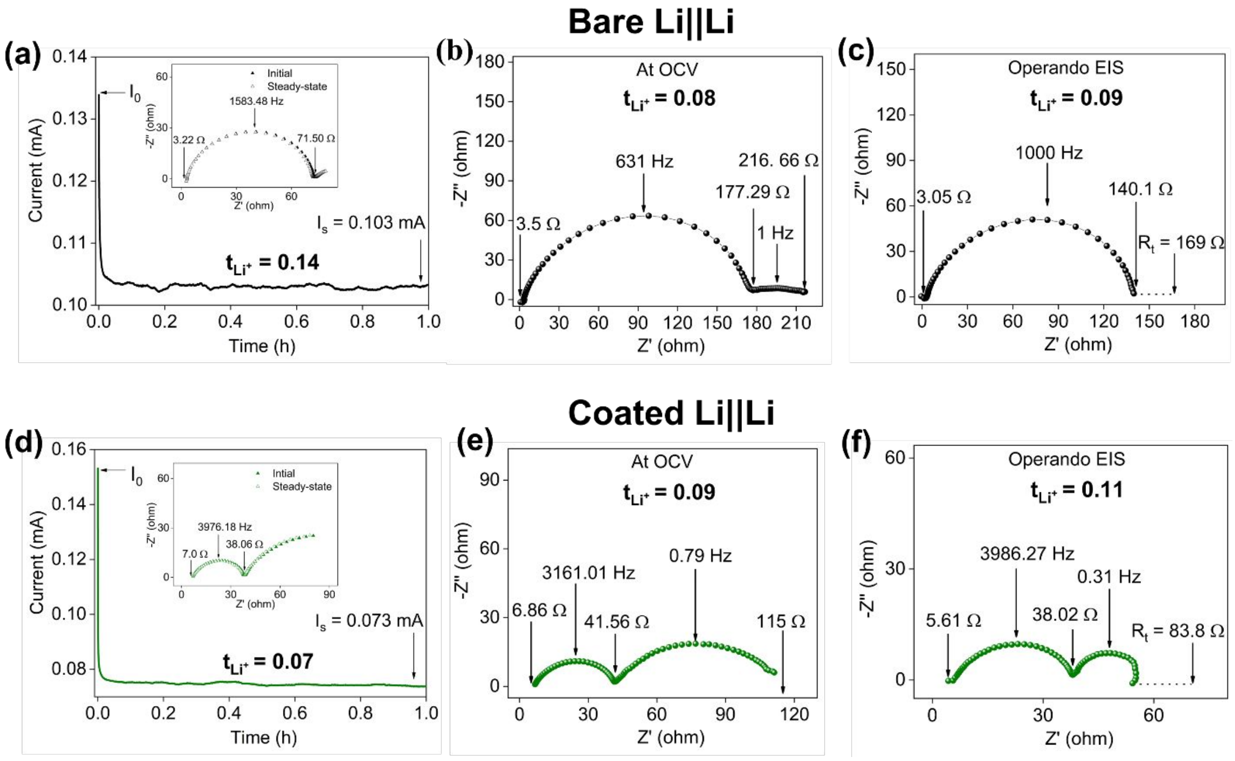

Figure S6. Transference number determination for bare Li cells (a-c) and coated Li cells (d-f) using the polarisation method, low-frequency measurement at OCV, and operando EIS (left to right).

Note 2: *Operando* EIS study of Li metal stripping and plating

To investigate the factors contributing to the improved cycling stability of coated lithium metal electrodes, we conducted operando electrochemical impedance spectroscopy on symmetric Li||Li cells with both bare and coated electrodes. This technique enabled us to correlate real-time impedance changes with the evolution of overpotential during galvanostatic strippingand plating. In the first half-cycle, lithium is stripped from the counter electrode and deposited onto the working electrode. Due to passivation, some of the deposited lithium reacts with the electrolyte, forming a solid electrolyte interphase (SEI) layer. During the reverse (charging) phase, this lithium is stripped and redeposited onto the counter electrode. Since both electrodes contain a large excess of lithium metal, the durations of stripping and plating remain constant. However, once the deposited lithium—partially lost to side reactions—is fully consumed, further stripping occurs via pitting corrosion from the bulk electrode. This process results in distinct overpotential trends depending on the cycle number. In the first half-cycle, the overpotential drops sharply and stabilizes at approximately half its initial value. In subsequent cycles, the behavior becomes more complex, exhibiting an initial peak, followed by a decrease, and then a gradual rise (Fig. 3b). Figure 4 presents the impedance spectra as stacked Nyquist plots rotated by 90°, with the low-frequency data points precisely aligned with the simultaneously measured overpotential values (shown in red and green). The plot configuration maintains consistent scaling between the imaginary impedance component (top axis) and the real impedance component (right axis), while the overpotential scale (left axis) directly correlates with the real impedance axis when accounting for the applied DC current amplitude.

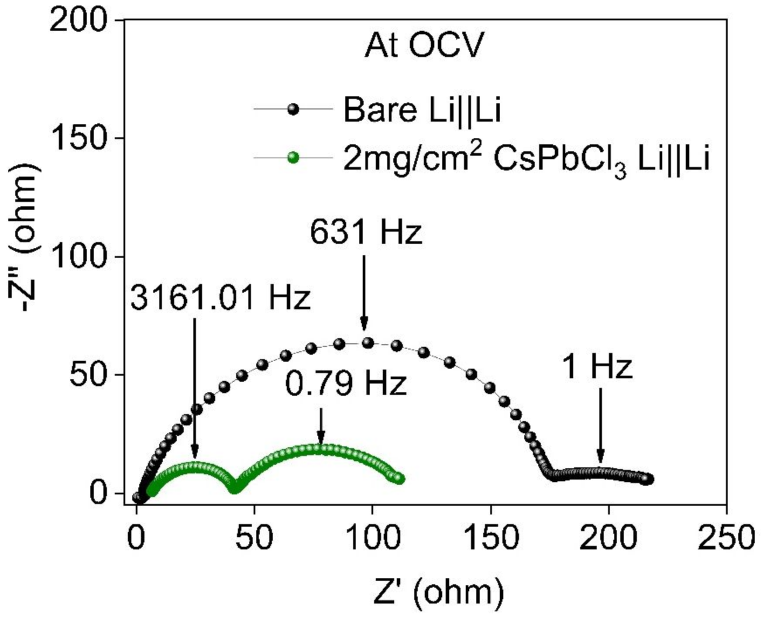

Figure S7. EIS spectra of bare and $CsPbCl_3$-coated Li||Li symmetric cell at OCV.

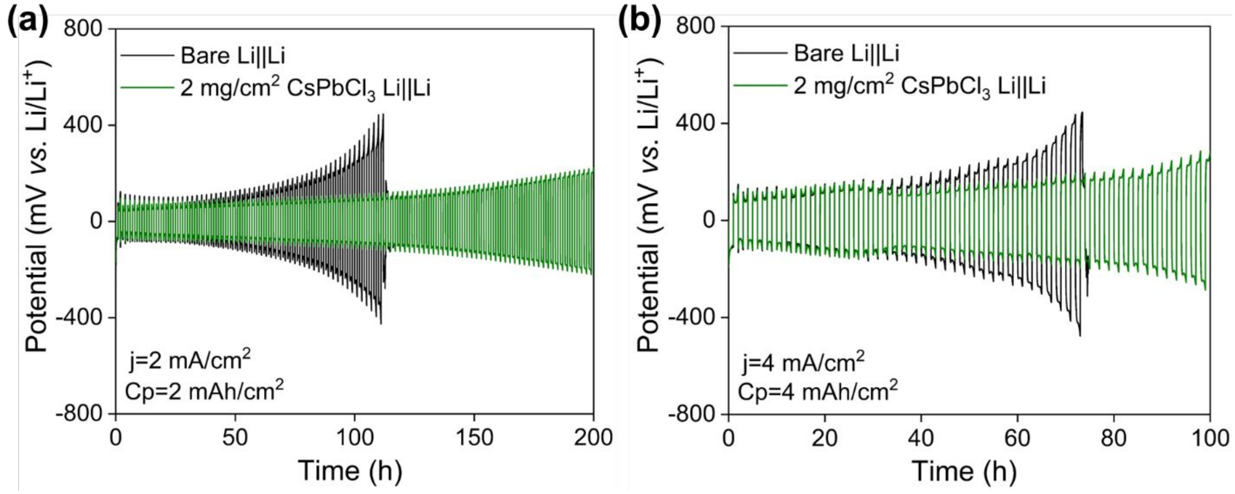

Figure S8. Galvanostatic cycling of bare and coated Li||Li symmetric cells at (a) areal capacity of 2 mA h cm^{-2} and a current density of 2 mA cm^{-2}, and (b) areal capacity of 4 mA h cm^{-2} & a current density of 4 mA cm^{-2} in 1 M LiFSI in FEC: DEC (1:2)

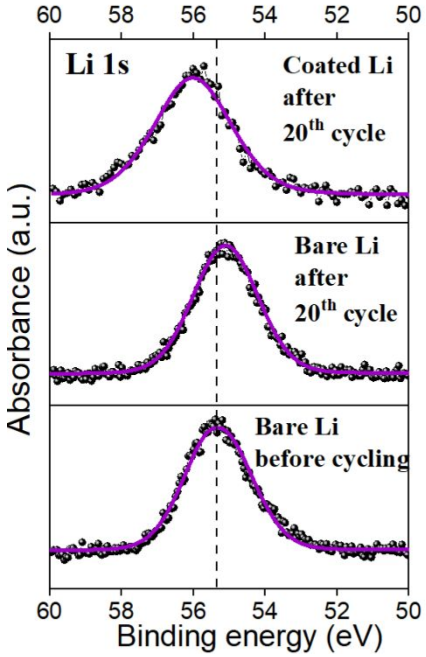

Figure S9. XPS spectra of the Li 1s of Bare Li before cycling (at the bottom), bare Li after cycling (in the middle), and $CsPbCl_3$ coated Li after cycling (at the top).

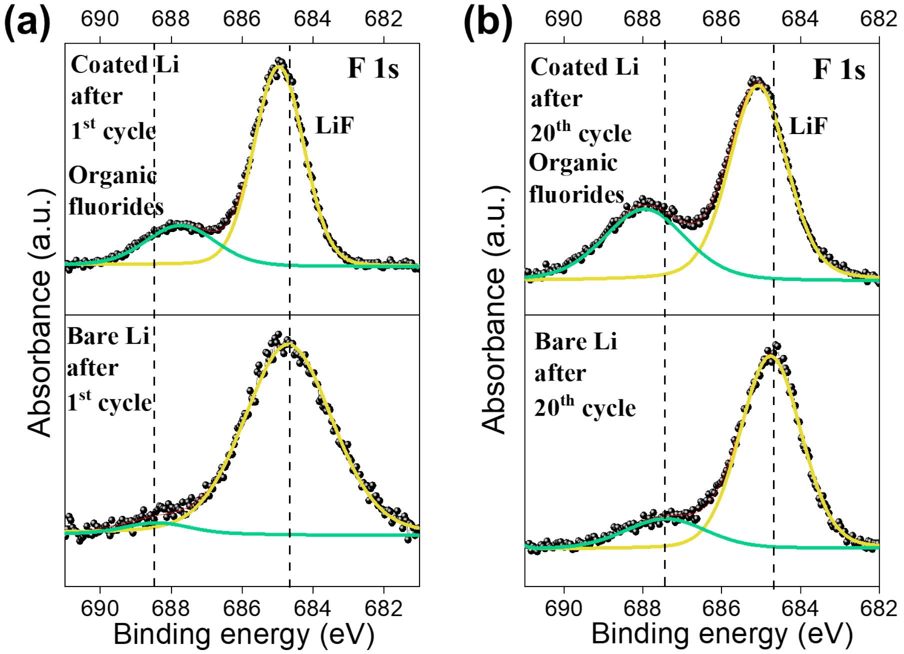

Figure S10. XPS spectra of the F 1s of $CsPbCl_3$ coated Li and bare Li after cycling (a) 1st cycle (b) 20th cycle

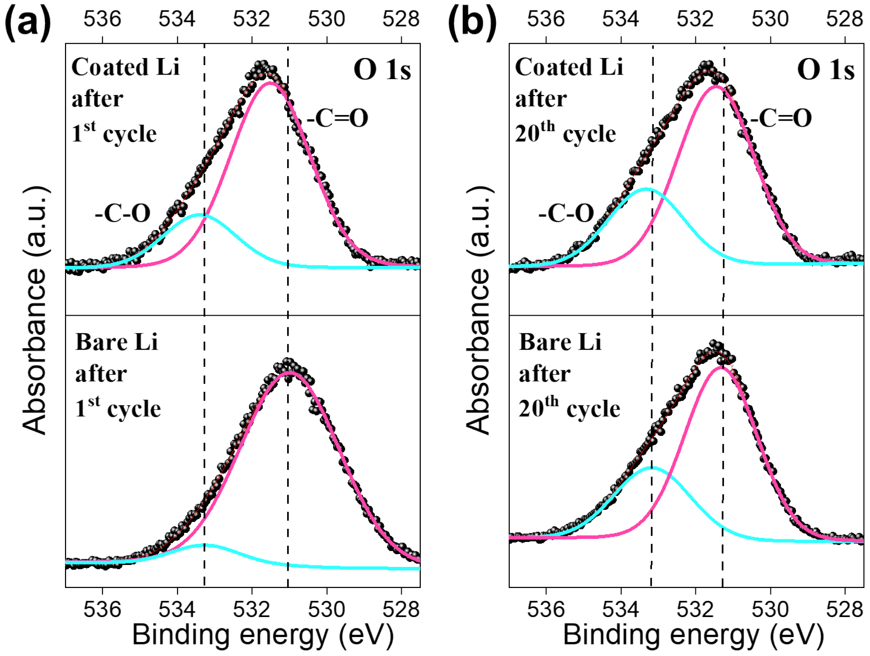

Figure S11. XPS spectra of the O 1s of $CsPbCl_3$ coated Li and bare Li after cycling (a) 1st cycle (b) 20th cycle

Table S1. Comparison of electrochemical performance of inorganic protective coatings for Li-metal anodes

Protective layer	Electrolyte	Li||Li cell performance	Full-cell performance	Ref.
$CsPbI_3$	1 M LiTFSI in DOL:DME (1 : 1)	1000 h at a current density of 1 mA cm^{-2} and discharge capacity of 1 mA h cm^{-2}	Li||S : 100% Coulombic efficiency throughout 1000 cycles at a 1C rate	[2]
$KNiF_3$	1.0 M LiTFSI in a mixture solvent of DOL/DME (1:1 by volume) & LP 30	3000 h at a high capacity of 4 mA h cm^{-2}	Li||LFP : Stable up to 120 cycles at 0.5C	[3]
$MASnCl_3$	1M $LiPF_6$ in EC:DME (1:1) + 1 vol% FEC	800 h at a current density of 1 mA cm^{-2} and discharge capacity of 1 mA h cm^{-2}	Li||LTO : Stable up to 500 cycles at 5C	[4]
$MAPbCl_3$	1M $LiPF_6$ in EC:DME (1:1) + 1 vol% FEC	600 h at a current density of 1 mA cm^{-2} and discharge capacity of 1 mA h cm^{-2}	Li||LCO : 85 % capacity retention after 100 cycles at 0.5C	[4]
Li–$CsPbCl_3$	1.0 M LiTFSI in DME : DOL (1:1) with 1 wt% $LiNO_3$ additive	1300 h under high current density of 10 mA cm^{-2}	Li||LFP : 91.4% after 230 cycles at 3C.	[5]
Li_2S_x (x=1, 2)	1.0 M LiTFSI in DME : DOL (1:1)	600 h under current density of 5.0 mA cm^{-2} and discharge capacity of 10.0 mAh cm^{-2}	Li||LFP : 88.0% capacity retention rate after 700 cycles at 1C	[6]
LiF@Li-zinc (Zn)	NASICON-type $Li_{1.5}Al_{0.5}Ge_{1.5}(PO)_4$ (LAGP)	1000 h at a current density of 0.1 mA cm^{-2}	Li||LFP : Reversible capacity (>150 mA h g^{-1}) after 40 cycles under 0.1 C	[7]
$CsPbCl_3$	1M LiFSI in FEC:DEC (1:2)	600 h at a current density of 1 mA cm^{-2} and discharge capacity of 1 mA h cm^{-2}	Li||LFP : 99.46 % capacity retention after 250 cycles at 1C	This work

REFERENCES

1 E. Barsoukov and J. R. Macdonald, Eds., *Impedance Spectroscopy*, Wiley, 2005.

2 N. Kaisar, A. Singh, P.-Y. Yang, Y.-T. Chen, S. Li, C.-W. Pao, S. Jou and C.-W. Chu, Long-lifespan lithium–metal batteries obtained using a perovskite intercalation layer to stabilize the lithium electrode, *J. Mater. Chem. A*, 2020, **8**, 9137–9145.

3 Y. Zhang, Y. Liu, J. Zhou, D. Wang, L. Tan and C. Yi, 3D cubic framework of fluoride perovskite SEI inducing uniform lithium deposition for air-stable and dendrite-free lithium metal anodes, *Chem. Eng. J.*, 2022, **431**, 134266.

4 Y.-C. Yin, Q. Wang, J.-T. Yang, F. Li, G. Zhang, C.-H. Jiang, H.-S. Mo, J.-S. Yao, K.-H. Wang, F. Zhou, H.-X. Ju and H.-B. Yao, Metal chloride perovskite thin film based interfacial layer for shielding lithium metal from liquid electrolyte, *Nat. Commun.*, 2020, **11**, 1761.

5 R. Liu, W. Feng, L. Fang, H. Deng, L. Lin, M. Chen, J.-X. Zhong and W. Yin, An ultrathin Li-doped perovskite SEI film with high Li ion flux for a fast charging lithium metal battery, *Energy Adv.*, 2024, **3**, 2999–3006.

6 J. Di, J. Yang, H. Tian, P. Ren, Y. Deng, W. Tang, W. Yan, R. Liu and J. Ma, Dendrites-Free Lithium Metal Anode Enabled by Synergistic Surface Structural Engineering, *Adv. Funct. Mater.*, DOI:10.1002/adfm.202200474.

7 J. Yu, Q. Liu, X. Hu, S. Wang, J. Wu, B. Liang, C. Han, F. Kang and B. Li, Smart construction of multifunctional Li1.5Al0.5Ge1.5(PO4)3|Li intermediate interfaces for solid-state batteries, *Energy Storage Mater.*, 2022, **46**, 68–75.
